# *Cinemotion*, a Program of Cognitive Remediation to Improve the Recognition and Expression of Facial Emotions in Schizophrenia: A Pilot Study

**DOI:** 10.3389/fpsyt.2018.00312

**Published:** 2018-07-23

**Authors:** Jessica Sevos, Anne Grosselin, Michael Gauthier, Florian Carmona, Aurélia Gay, Catherine Massoubre

**Affiliations:** ^1^Department of Psychiatry, University Hospital of Saint-Etienne, Saint-Etienne, France; ^2^TAPE Laboratory, EA 7423, University of Jean Monnet, Saint-Etienne, France; ^3^Referal Center of Psychosocial Rehabilitation, Saint-Etienne, France

**Keywords:** schizophrenia, social cognition, cognitive remediation, recognition of facial emotion, expression of facial emotion, embodied simulation

## Abstract

**Objective:** Patients with schizophrenia exhibit impaired social cognition, especially in the recognition and expression of facial emotions, aspects of communication profoundly interlinked in an embodied approach of cognition. Nevertheless, many training programs have been developed that focus on either of these deficits but not both. We therefore designed a training program, *Cinemotion*, intended to remedy the 2 deficits and investigated its feasibility and effects in patients with schizophrenia.

**Design:** Thirty-one patients undergoing treatment for schizophrenia and presenting deficit in emotion recognition were randomized to a group of 16 to undergo Cinemotion training, delivered in weekly group sessions, and to a control treatment group of 15. At the conclusion of training or after 10 weeks in controls, we reassessed and compared original and final results to determine improvement.

**Methods:** Facial emotions recognition (TREF), empathy (Questionnaire of Cognitive and Affective Empathy, QCAE), and attributional style (Ambiguous Intentions Hostility Questionnaire, AIHQ) were assessed before (T0) and after (T1) the program. External evaluators also assessed ability and accuracy of Cinemotion participants to self-generate facial emotion expression in response to verbal instruction.

**Results:** Between T0 and T1, Cinemotion participants significantly improved total TREF, sadness, disgust, and anger scores, compared to findings in control treatment group. They also improved their ability and accuracy to self-generate facial expressions, especially sadness and fear, with no significant improvement in other components of social recognition.

**Conclusions:** Our findings show the apparent efficacy of training using the *Cinemotion* program to improve the recognition and expression of facial emotions in schizophrenia.

## Introduction

Social cognition is a multidimensional construct that comprises the “psychological processes (…) involved in the perception, encoding, storage, retrieval and regulation of information about other people and ourselves” [(1); pp. 620] and encompasses 4 core domains: the processing of emotions, social perception, theory of mind (ToM), and attributional style (2). Some authors also include empathy as a fifth domain (3).

Many studies highlight the frequent manifestation of major deficits in social cognition in schizophrenia (4, 5). Often, these disorders are present at the beginning of the disease in the subjects at risk, and worsen as the disorders evolve and the social links deteriorate. As they can be considered as the strongest predictor of functional outcome in this illness (6), many interventions to treat deficits in social cognition have been proposed in recent years [see (7) for review], and these span a wide diversity of therapeutic modalities, including targeted interventions focused on a single social cognitive ability, such as Training of Affect Recognition (TAR) (8) and Theory of Mind Intervention (ToMI) (9), or broad-based interventions, such as Social Cognition and Interaction Training (SCIT) (10). Such focused interventions have seemed particularly promising in improving social cognition (11), yielded moderate to large effects with respect to the processing of emotion (mostly recognition of facial emotion) as well as theory of mind, and moderate effects associated with social perception and attributional style (12).

### Recognition of facial emotion

Patients with schizophrenia demonstrate a relatively large deficit in the ability to recognize the facial expressions of others (13), but its precise nature remains unclear (14). Different hypotheses have speculated such causes as attentional deficit (15), atypical pattern of visual exploration of the face (16), or deficit in the configural processing of informations (17, 18). One review of the literature highlights the inferior recognition of almost all emotions among patients with schizophrenia compared to that of healthy subjects (17), with the severity of this deficit differing according to the specific emotion under consideration. For example, subjects with schizophrenia might recognize expressions of disgust (17) or fear (19) less well than other emotions, but expressions of joy equally well than controls.

Deficiency in facial recognition shows low sensitivity to antipsychotic treatments (13) and can lead to a poor social functioning (8) or a social withdrawal (20), so researchers have investigated various methods to remedy difficulties in emotion recognition in schizophrenia.

Wölver et al. (8) developed a program of Training Affect Recognition (TAR), which combines computer-assisted, paper-and-pencil and home exercises, and involves 2 patients with schizophrenia working in collaboration with a therapist during 12 one-hour sessions over 2 weeks. The 12 sessions of the TAR program are divided into 3 blocks of 4 sessions each. The first block focuses on the identification and discrimination of the prototypical facial signs of 6 emotions considered basic and universal and utilizes verbalization. The second is directed at integrating this initial piecemeal approach to the recognition of facial emotion into a more holistic mode of treatment, which requires quick decision-making based on first impression and nonverbal processing, and that addresses facial expressions with small intensity. The third block targets the processing of the non-prototypical and ambiguous expression of emotion often encountered in everyday life and integrates facial expression into the situational context. The TAR program has known effects on facial affect recognition, particularly sadness, and on the social relationship domain associated with quality of life (8). More recently, however, Wölver and Frommann (21) observed that the TAR program may be less targeted than initially intended, noting significant improvements in prosodic affect recognition, ToM, and social competence with TAR training.

In France, the GAÏA schizophrenia facial affects recognition cognitive enhancement program (GAÏA s-face) (22) is the only cognitive remediation therapy that focuses on the recognition of facial emotions. GAÏA s-face is a computer-assisted program that offers individualized treatment during 30 sessions, consisting of 3 one-hour sessions per week over 10 weeks divided into 3 phases. In the first phase, patients complete exercises utilizing photographs that allow them to establish and verbalize the criteria on which they rely to recognize and differentiate the emotions of joy, anger, and sadness. In the second phase, the patient engages in computerized exercises in which they view video sequences and then answer questions that require them to adapt strategies developed in the photograph exercises to non-static situations. In the third phase, learning associated with joy, anger, and sadness is generalized to other basic emotions (fear, disgust, and contempt) and some complex emotions.

In a more recent study, Gaudelus et al. (23) compared the GAÏA s-face program with the RECOS program of neurocognitive remediation therapy that focuses on selective attention (24), and they utilized subject performances on the test of facial emotion recognition (TREF) (22) as the main measure of treatment outcome. They showed significantly improved recognition of facial emotion in the GAÏA group but also in RECOS group (with a significantly smaller effect), no effect of social cognitive remediation on ToM, attributional style, or empathy in either group, and significantly improved scores of social autonomy (EAS, [Echelle d'Autonomie Sociale]; social autonomy scale) (25) in the GAÏA group only.

### Expression of facial emotion

Individuals process emotions at 3 levels, recognizing the emotions of others and experiencing and expressing their own emotions, whereas those with schizophrenia demonstrate limited recognition of others' facial expressions in addition to disturbances in their own facial expressivity and experienced emotion. For instance, one study reported that during the viewing of happy, sad, scary, or neutral film extracts, patients with schizophrenia and controls found the content of the films equally emotional but physiological measures of arousal (assessed by cutaneous conductance responses) were greater among those with schizophrenia, and they displayed fewer expressions (26). More specifically, patients with schizophrenia have been shown to manifest fewer positive and negative facial expressions than healthy subjects in response to emotional stimuli, especially those that involve the upper part of the face (27).

Using the Facial Expression Coding System [FACES; (28)], other studies that have included photographs or video recordings have shown significant disturbance in simulated (29) and evoked expressions (30). In addition, less accurate production of facial expressions has been reported in patients compared with controls in response to verbal instructions in all emotion conditions (31). These authors surmised that that situation might be attributed to possible deficits in the integration of sensory and motor inputs, such as those suspected to affect motor representations from sensory input that underlie the inability to imitate facial expressions in patients with schizophrenia.

Park and associates (32) also described a fundamental impairment of the ability to imitate in schizophrenia that could lead to difficulties in mind reading. They noted that imitation has “biological and social significance,” that individuals internalize and simulate the actions of others to understand their meaning, thereby suggesting that a basic deficit in the ability to imitate could interfere with the internalizing, simulating, and understanding of the emotions and actions they observe (33) and their inference of the intentions and beliefs of others. Gallagher and Varga (34) aligned these results with dysfunction in the mirror neuron system of patients with schizophrenia (35), demonstrating that a rehabilitation strategy based on both observation and imitation could improve the ability of these patients to make inferences about other people's emotions and mental states.

### Embodied simulation

In agreement with Adolphs and associates (36), we believe that the individual recognizes the emotional state of another by generating on-line internal somatosensory representations based on his own prior experience (37, 38), that simulate how the observer would feel when expressing the same given facial emotion (39). Thus, one's visual recognition of facial expressions would involve the simultaneous processing of the visual representations of the perceptual properties of the facial expressions with somatosensory simulation of the emotion associated with these expressions. That is, it would be as if the observer performed the same emotions as the individual observed, a process described as “embodied simulation” (35, 40). These ideas are also supported by findings of neuroimaging studies that reveal that when we observe others' emotional states, we activate the same neural systems (the mirror neuron system) activated by our own emotions (41, 42). Recognition of emotions, therefore, requires the simulation of body states observed visually, that is, reactivation of the circuits involved in past learning of similar emotional situations, even if the subject does not want to imitate the emotion (34). Some authors suggest impairment of the capacity of those with schizophrenia to spontaneously simulate another person's subjective world (32, 34, 43). Indeed, their reduced abilities to express facial emotions may provide them with fewer proprioceptive cues when they attempt to recognize or understand the emotions of others (44). For Kring and associates [(45), p. 187], if “*…pictures of facial expressions elicit similar facial reactions in observers, and these reactions may aid in the recognition and perception of emotion depicted in the faces*”, promoting facial emotion expression may impact facial affect recognition in schizophrenia and should be incorporated into current social skills training programs (44). However, compared to the relatively larger amount of training on facial recognition, there are very few and pilot studies addressing specifically facial expression of emotions.

### The present research

In the light of the programs described above, we developed a short and entertaining French program to improve both the recognition and expression of facial emotions in patients with schizophrenia utilizing extracts of motion pictures, and we have called our program *Cinemotion*.

The program is organized into 4 phases in which the patient: (1) identifies the main emotion expressed in cinematographic extracts (46); (2) implements strategies; (3) validates and reinforces the learned strategies; and (4) expresses the facial emotion trained during the session.

*Cinemotion* focuses on 3 important points: the use of motion picture extracts rather than static photographs to simulate actual everyday life; group rather than individual work to favor interactions among participants, thereby introducing new and different coping strategies and encouraging the transfer and adaptation of the strategies into real-life situations; and modeling of the expressive component of facial emotions to provide patients more proprioceptive signals essential to the recognition of these emotions in others.

In the present study, we investigate the effects of training using the *Cinemotion* program and its feasibility for the remediation of both impaired recognition and expression of facial emotions in patients with schizophrenia.

## Materials and methods

### Participants

Participants were 31 patients with stable schizophrenia (4 in- and 27 out-patients). Inclusion criteria were: DSM-V (47) diagnosis of schizophrenia, clinical stability confirmed by their referring psychiatrist (i.e., symptoms at a low level and stable, no psychiatric hospitalizations in the past 6 months and same antipsychotic medication for past 3 months), age between 18 to 55 years, native French speaker, no change in antipsychotic medication and clinical status within the 4 weeks prior to the study, IQ score above 70 (48), and impairment of facial emotions recognition confirmed by scores on a TREF test (for at least one emotion other than contempt).

The local ethics committee of the University Hospital of Saint-Etienne approved the study, and patients, who were all volunteers, provided written consent to participate after the nature of the procedure was fully explained.

### Design

We randomly assigned 16 patients (5 women) to participate in the *Cinemotion* training and 15 patients (3 women) to be controls with their usual treatment. Two in-patients were included in each group. The usual treatment included regular hospital care that could consist of individual and/or group psychotherapy, therapeutic education, or occupational art workshops. None of the participants received social- or neurocognitive-oriented treatment during or 6 months before the study period.

Prior to the beginning of Cinemotion training (T0), all 31 underwent assessments of/by: intelligence quotient [IQ]; positive and negative syndrome scale [PANSS]; Scale for Assessment of Negative Symptoms [SANS]; facial emotions recognition test [TREF]; Questionnaire of Cognitive and Affective Empathy [QCAE]; and Ambiguous Intentions Hostility Questionnaire [AIHQ] (see descriptions below). They underwent additional testing (TREF, QCAE, AIHQ) at T1, that is after the Cinemotion program for the Cinemotion group and after 10 weeks of their usual treatment for the Control treatment group, in order to assess the effects of *Cinemotion* training.

At T1, a group of 10 raters scored ability and accuracy of Cinemotion participants to self-generate facial emotion expression in response to verbal instruction, on video recorded at the end of each session. The ratings of their performance between the first and second training session for each emotion were compared.

### Interventions

*Cinemotion* is a program for the remediation of social cognition that focuses on the recognition of facial emotion and expression of the 5 basic emotions of joy, sadness, fear, anger, and disgust (49). Three to seven participants undergo training once a week for 90 min for 10 weeks, and 2 therapists (combination of neuropsychologist, resident in psychiatry, or nurse) animate. Each basic emotion is trained during 2 sessions via 3 different film extracts per session, and each session is organized into 4 distinct phases. In the first phase, the subjects view cinematographic extracts linked to specific emotions and are asked to identify the main emotion, which requires them to both interpret the social and emotional context of the scene and recognize the facial expressions of the characters. In the second phase, they analyze the facial expressions with a verbal description of the specific aspects of the emotion, and they isolate contextual clues (light, sound context, scenario, body movements, and prosody) of the scene. This phase aims to develop better strategies for extracting relevant information in a global and ecological situation, with particular focus directed at the crucial parts of the face (eyes, mouth, and nose). During this phase, the participants are also invited to interact with each other, to share their subjective and bodily experiences and expression as aroused by the extracts. In the third phase, subjects re-view the same extract to validate and reinforce the learned strategies. The 3 preceding phases are repeated for the 3 extracts trained during each session. After all 3 phases are complete for all 3 video extracts, in the 4th phase, the therapists simulate and demonstrate the facial emotion trained during the session, and participants are asked to express the emotion in front of a camera, and video recordings are made.

The next session begins with the viewing and discussion of the video recordings of the participants' facial expressions from the previous session.

Throughout the sessions, the exercise becomes more complex as the intensity of emotion decreases and the limits distinguishing the different emotions dwindle.

As part of the remediation, we selected extracts of quality films according to their direct link with a basic emotion from the database proposed by Schaefer et al. (46). Spoken language of all extracts was French. We removed the emotion “contempt” from our remediation program because it was not offered in the data bank and selected 6 extracts of each of the other basic emotions for the remediation training. We used a computer device with video projector to present the extracts and a video camera to record the exercises performed at the end of each session.

### Assessments

#### Facial emotion recognition

The main measure of treatment outcome was performance on the TREF ([Test de Reconnaissance des Emotions Faciales]; facial emotions recognition task) (50), which evaluates the ability to recognize facial emotions and consists of 54 pictures that represent 6 basic emotions (joy, anger, fear, contempt, disgust, and sadness) at 9 levels of intensity (from 20, 30, …to 100%). The subject views each photograph for 10 s and selects the perceived emotion from a list of the six. This test provides an overall score of emotion recognition, a subscore by emotion, and their threshold of detection.

#### Self-generation of facial emotion expression

At the end of each session, participants were asked to self-generate facial expression related to the emotion trained during the session (joy during Sessions 1 and 2, sadness during Sessions 3 & 4, …) in front of a camera, and a PowerPoint® slide show was created by randomly mixing the videos of the therapist's and participant's expressions from the 2 training sessions for each emotion. We asked 10 individuals (nurses who did not know the patients enrolled in the study) to categorize each video expression as disgust, joy, fear, sadness, or anger, on the basis of the appearance of facial features associated with particular emotions. In case of error, the correct answer was given by the experimenter and then, they were asked to rate the accuracy of each participant's expression using a 7-point Likert-type scale (1, not at all accurate; 2, barely accurate; 3, slightly accurate; 4, somewhat accurate; 5, reasonably accurate; 6, highly accurate; and 7, perfectly accurate).

#### Clinical assessments

At T0, all patients were assessed using the Positive and Negative Syndrome Scale [PANSS; (51)] and the Scale for the Assessment of Negative Symptoms [SANS; (52)] by the same senior psychiatrist.

#### Cognitive assessments

Attributional style was assessed using the Ambiguous Intentions Hostility Questionnaire-Ambiguous items [AIHQ-A; (53)]. In this task, subjects read 5 short vignettes that describe negative interpersonal events with ambiguous causality and are then asked to answer 3 open questions related to hostility (“*Why did the other person do what s/he did*?”), aggression (“*How would you respond*?”), and blame (“*How much would you blame the person*?”). Scores on each range from 0 to 5, and the sum in each category permits calculation of scores of hostility (HB) and aggression (AB) biases and “blame.” Higher scores indicate greater bias.

Empathy was assessed with the Questionnaire of Cognitive and Affective Empathy [QCAE; (54)], in which subjects read 31 items that they rate on a 4-point Likert-type scale (with the response options strongly agree, slightly agree, slightly disagree, and strongly disagree), the sums of which yield scores of cognitive (CE) and affective (AE) empathy.

### Statistical analysis

We analyzed statistics using SPSS 18.0 software. Group differences in demographic and clinical characteristics were analyzed using the *t*-test for independent samples or the chi-squared test for qualitative values. To account for any between-groups differences at pretreatment, we performed an analysis of variance (ANCOVA) in participant data using T1 scores as dependent variables, group as a fixed factor, and T0 scores as covariates. To characterize the magnitude of treatment effects, effect size estimates are presented for both between-group differences using Cohen's partial eta squared (${\text{n}}_{\text{p}}^{2}$) and within-group pre- to post-treatment changes using Cohen's d (55). Effect sizes presented as ${\text{n}}_{\text{p}}^{2}$ correspond to the following conventions: small (0.01), medium (0.06), and large (0.14). Effect sizes presented as d correspond to the following conventions: small (0.20), medium (0.50), and large (0.80). *P* < 0.05 was considered statistically significant.

## Results

The *Cinemotion* group seemed to accept the program well based on the attendance of all participants in at least 7 of the 10 sessions (85% mean session attendance).

### Participants

The *Cinemotion* and control groups did not differ in age, education, duration of illness, IQ, or clinical characteristics (see Table 1). There was no sex prevalence between groups (χ^2^ = 0.512; *P* = 0.474).

**Table 1:** Means (*SD*) comparisons of demographic and clinical characteristics of the *Cinemotion* and control groups.

	***Cinemotion* (*N* = 16)**	**Control (*N* = 15)**	***t*-test; *P* value**
Age (years)	39.8 (10.3)	42.7 (5.6)	*t =* −0.967, *P* = 0.342
Education (years)	10.9 (3)	10.7 (2.4)	*t* = 0.211, *P* = 0.834
Illness duration (years)	12.4 (7.5)	17.9 (7.1)	*t =* −2.040, *P* = 0.051
IQ	95.8 (10.8)	97.7 (7.0)	*t =* −0.574, *P* = 0.570
PANSS positive	12.9 (3.3)	12.7 (4.9)	*t* = 0.137, *P* = 0.892
PANSS negative	17.6 (6.3)	17.5 (5.9)	*t* = 0.044, *P* = 0.965
SANS	40.13 (16.6)	37.2 (15.9)	*t* = 0.512, *P* = 0.613

*IQ, Intelligence Quotient; PANSS, Positive And Negative Syndrome Scale; SANS, Scale for Assessment of Negative Symptoms*.

### Assessments

#### Facial emotion recognition (TREF)

Between T0 and T1, the total TREF, disgust, sadness and anger scores showed significant improvement among Cinemotion participants compared to those of controls (see Table 2). For all of these emotions, the magnitude of the between-group effect was large. While the within-group effects were large for total TREF and disgust in both groups, we observed a medium to large increase for sadness and large increase for anger in the Cinemotion group and small decrease for sadness and small to medium increase for anger in the control group.

**Table 2:** Evolution of mean scores (± standard deviation [SD]) and range of emotion recognition (TREF), attributional style (AIHQ), and empathy (QCAE), according to group (*Cinemotion* or Control) and time of evaluation before (T0) or after (T1) the training program.

	**Cinemotion**		**Control**		***F*-test**	**Effect sizes**		
	**T0**	**T1**	**T0**	**T1**		**Between group (${\text{n}}_{\text{p}}^{2}$)^a^**	**Within cinemotion group (d)^b^**	**Within control group (d)^b^**
**TREF**								
Total	57.2 (7.9)	73.8 (6.1)	54.8 (8.8)	64 (8.3)	13.025^**^	0.317	1.4	1.11
	41–67	63–87	37–67	46–76				
Disgust	44.4 (21.1)	69.4 (11.1)	42.2 16.4)	59.3 (14.3)	4.632 ^*^	0.142	1.01	1.01
	0–78	44–89	11–67	33–78				
Contempt	33.3 (18.1)	40.3 (18.1)	22.2 (15.1)	37.8 (18.7)	0.003	0.001	0.32	0.72
	11–67	11–67	0–56	11–78				
Joy	87.5 (10.6)	93.1 (8.0)	91.9 (7.8)	96.3 (6.9)	1.013	0.035	0.43	0.48
	67–100	78–100	78–100	78–100				
Fear	68.1 (20.2)	87.5 (9.0)	73.3 (16.7)	85.2 (14.9)	0.613	0.021	0.85	0.87
	33–89	67–100	44–100	56–100				
Sadness	65.3 (15.1)	78.5 (15.4)	64.4 (21.1)	61.5 (26.2)	6.912^*^	0.198	0.72	−0.17
	44–89	56–100	33–100	11–89				
Anger	50.7 (16.7)	74.3 (13.3)	34.8 (23.7)	43.7 (14.2)	30.075^**^	0.518	1.42	0.51
	11–78	44–100	0–67	33–67				
**AIHQ**								
HB	2 (0.9)	1.8 (0.6)	2.1 (0.9)	1.9 (0.8)	0.149	0.005	0.39	0.27
	1–4.2	1–3	1–3.8	1–4				
Blame	2.7 (1.2)	2.4 (0.9)	2.8 (0.9)	2.6 (0.8)	0.219	0.008	0.49	0.42
	1–4.5	1–4.1	1.3–4.3	1.1–4				
AB	1.6 (0.5)	1.4 (0.5)	1.5 (0.6)	1.4 (0.4)	0.141	0.005	0.32	0.08
	1–2.6	1–1.8	1–3.4	1–2.2				
**QCAE**								
Total	80.9 (13.2)	84.8 (11.6)	82.5 (10.4)	87 (7.4)	0.263	0.009	0.53	0.44
	61–109	62–108	64–98	74–99				
CE	48.8 (9.6)	51.9 (8.9)	51.4 (6.8)	53.7 (6.5)	0.001	0.001	0.43	0.54
	36–70	38–72	38–65	42–64				
AE	32.2 (5.4)	32.9 (5.3)	33.1 (5.4)	33.3 (4.8)	0.021	0.001	0.15	0.09
	18–40	24–40	25–41	25–41				

*F-tests are based on ANCOVA's using T1 scores as dependent variables, group as a fixed factor, and T0 scores as covariates*.

*AB, aggressive bias; AE, affective empathy; AIHQ, Ambiguous Intentions Hostility Questionnaire; C, contempt; CE, cognitive empathy; HB, hostility bias; QCAE, Questionnaire of Cognitive and Affective Empathy*.

a *Positive effect sizes indicate greater improvement in the Cinemotion group*.

b *Positive effect sizes indicate improvement from T0 to T1*.

* *p ≤ 0.05*;

** *p ≤ 0.001*.

At T1, TREF scores of those receiving *Cinemotion* training indicated they no longer demonstrated deficits in the recognition of joy, fear and sadness (see Table 3), and only 6% still showed deficits in total TREF and anger scores. In contrast, controls still scored poorly in overall recognition of emotion (47%) and in the recognition of anger (67%). Sixty-three percent of the *Cinemotion* participants still showed deficits in the recognition of contempt because it was not trained during the remediation program.

**Table 3:** Proportion (%) of patients who exhibited some deficits at first (T0) and second (T1) testing of facial emotions recognition (TREF).

	***Cinemotion***		**Control**	
	**T0**	**T1**	**T0**	**T1**
Total	75	6	80	47
Disgust	56	6	67	20
Contempt	44	63	80	53
Joy	6	0	0	0
Fear	31	0	33	13
Sadness	19	0	26	33
Angry	38	6	73	67

#### Self-generation of facial emotion expression

Agreement between raters' scores was high (0.94), as assessed by intraclass correlation [ICC; (56)]. Table 4 displays the average rating for each expression produced in response to verbal instruction in Sessions 1 and 2.

**Table 4:** Mean ratings for self-generated expressions in the first and second sessions for each trained emotion in the *Cinemotion* group.

	**Categorization**			**Accuracy**		
	**Session 1**	**Session 2**	**Z-test; *P* value**	**Session 1**	**Session 2**	**Z-test; *P* value**
Total	78.5	85.5	Z = −2.081; *P* = 0.037	4.56	4.93	Z = −2.490; *P* = 0.013
Disgust	85	85	Z = 0.000; *P* = 1.00	5.38	4.92	Z = −1.667; *P* = 0.095
Joy	95	100	Z = −1.414; *P* = 0.157	5.48	5.88	Z = −1.904; *P* = 0.057
Fear	97.5	100	Z = −1.000; *P* = 0.317	4.75	5.33	Z = −2.322; *P* = 0.020
Sadness	67.5	95	Z = −2.428; *P* = 0.015	3.88	4.98	Z = −2.561; *P* = 0.010
Anger	47.5	47.5	Z = −0.108; *P* = 0.914	3.33	3.53	Z = −0.723; *P* = 0.469

Globally, the ability of *Cinemotion* participants to generate expressions, particularly sadness, improved from Session 1 to 2. Their accuracy improved in expression especially for fear and sadness, and we observed a marginally significant trend for disgust and joy conditions. Only their abilities to express the emotion of anger showed no improvement with respect to categorization (with very low recognition scores for this emotion at both times) and accuracy.

#### Cognitive assessments

There were not significant between-groups effects for the other cognitive assessments (AIHQ –attributional style- and QCAE –empathy- tasks) (see Table 2).

## Discussion

Our findings provide preliminary evidence of the feasibility of our new program of social cognitive skills training, *Cinemotion*, to improve recognition and expression of facial emotions among in- and outpatients with schizophrenia. Indeed, we understand that alterations in the treatment of emotions in schizophrenia affect both domains and that the two are profoundly interlinked, so it seems essential to offer patients a training program that focuses simultaneously on these 2 domains.

Overall scores of TREF and especially scores for disgust, sadness and anger improved significantly in *Cinemotion* participants, with large between-group effects. Their scores for the recognition of joy were very high before training and therefore did not improve significantly, probably because joy is known to be the simplest emotion to recognize (57, 58). Furthermore, we found no difference concerning contempt because we did not train for this emotion. In addition, though scores for fear improved for the *Cinemotion* participants between T0 and T1, they also improved for controls, which might explain an absence of significant results in between-group analysis. This test-retest effect was also observed for the total TREF score [as already reported by Gaudelus et al. (23) in their RECOS control group]. More qualitatively, *Cinemotion* participants no longer demonstrated deficiencies in their recognition of fear and sadness after the training, and only 6% still have difficulties with respect to anger. The significant improvement in TREF results for the *Cinemotion* group compared to the control treatment group is consistent with data from the literature showing that facial emotions recognition can be improved using a specific remediation (12).

Subjects with schizophrenia less accurately identify the facial expressions of others and are less expressive themselves (27, 31, 59). We found this deficit in facial expressiveness related to difficulties in the accuracy of their expression of the given emotion (that is, little resemblance to one of the 5 emotional expressions trained, or its intensity was too weak to be detected). However, the training allowed external evaluators to recognize and judge more accurately the emotions expressed by the participants, whose ability to self-generate facial expressions increased in response to verbal instruction (especially sadness and fear and marginally, disgust, and joy). Because the expression of facial emotions is a key element in interpersonal relationships, it is important that subjects with schizophrenia develop the ability to convey emotion nonverbally through facial expression. Our results also align with those of pilot studies that show that the practice of generating expressions can improve facial expressiveness (31), facial affect perception (44, 45), and the ability to take on another person's perspective (34) in patients with schizophrenia. Therefore, teaching of the accurate expression of facial emotions could be incorporated into current programs of social skills training (44). However, more experimental studies involving training in both the recognition and expression of facial emotion are needed to explore the directionality of these relationships. *Cinemotion* training was inspired by existing programs, and its strength lies precisely in this dual training (recognition and expression). Indeed, theories of embodied cognition postulate the fundamental grounding of cognitive processes in their physical context (60), so perception alone is insufficient to arouse or recognize emotion if the body states are not associated (61). Niedenthal et al. (42) suggested that perceiving and thinking about emotion involve perceptual, somatovisceral, and motoric re-experiencing of the relevant emotion in one's self, that emotion would exist only once incarnated (62). Problems of social cognition may be newly viewed as disordered embodied communication and embodiment perspective suggests novel treatment strategies through body-oriented interventions (63).

According to this theoretical approach, we felt it relevant to propose sessions in which patients could share their bodily feelings and try to express physically the trained emotions. Our preliminary findings have encouraged us to develop a new session format that includes the more precise evaluation (than Likert scales) of the overall dynamic facial changes during emotional expression (accuracy, intensity, and duration) using the Facial Expression Coding System [FACES: (28)], which identifies discrete facial muscle movements. For these sessions, self-generation of all emotions will be assessed at T0 and at the end of the training.

Tests related to other components of social cognition have not led to significant improvements, neither in *Cinemotion* group nor in control treatment group. Regarding the attributional style (AIHQ), this might reflect a ceiling effect, such as already described by Roberts et al. (10) for SCIT. Indeed, good results of patients before the program left little room for improvement. About empathy, assessed by QCAE, patient scores from our study are similar to those obtained by Michaels et al. (64). The absence of significant after-training improvement could be explained by the nature of the test. Indeed, Bonfils et al. (65) suggest that schizophrenic patients perceive themselves as more empathic than their actual empathic skills suggest. Furthermore, if through imitation and mimicry motor component of empathy contributes to both cognitive and affective processes (66), it might be interesting to use performance-based rather than self-report measures in the future. Furthermore, the greatest amount of training time and attention was directed at the recognition and expression of facial emotion. Nevertheless, other social cognition tests should be used in a next study. Such assessments include the Social Knowledge Test (SKT) (67); comic-strip task (68); Internal, Personal, and Situational Attributions Questionnaire (IPSAQ) (69).

Another particularity of the *Cinemotion* program is that it was designed to create and facilitate interactions between participants based on the assumption that linking patients with similar pathological features could promote acceptance and understanding of their disorders. Thus, patients could feel safe enough to interact with others, share their experience, discuss their feelings, present their understandings of the movie extracts and, working together, they did help each other establish strategies to recognize facial emotions. We can only imagine the positive impact of this interaction on the results of TREF and could also expect improvements in the other core domains of social cognition.

Participants found the *Cinemotion* training engaging, enjoyable, and relevant to their daily lives and described watching film extracts and “acting out” emotions as both workout and entertainment. Though some participants found expressing emotion in front of the camera an unprecedented experience and real challenge, attendance was very good.

Our project was limited because it was a pilot study of a small sample, so conclusions are tentative until replicated using larger and more controlled protocols that include assessments of facial muscle movements during the expression phase and more relevant scales of social cognition. Comparison with another remediation program, as control treatment balanced for frequency and intensity, and assessment of improvements over time to observe whether benefits are sustainable should also be interesting. Although the total duration of the *Cinemotion* group is comparable to other some programs [10 sessions lasting 90 min vs. 30 sessions lasting 30 min in the GAÏA s-face program—Gaudelus et al. (23)—or 12 sessions lasting 60 min in the TAR program—(8)—], it could be interesting to include two more sessions to rework all emotions previously trained. Finally, to help transfer therapeutic strategies into daily life, it could be relevant to integrate individualized homework tasks into the training program, in which participants should apply the learned skills to interpersonal problems in their own lives.

## Conclusion

Our *Cinemotion* program is uniquely designed to improve both the recognition and expression of facial emotions among in- and out-patients with schizophrenia by utilizing video recordings that reflect the physical, social, and environmental aspects of real-world interactions. This study provides preliminary evidence that *Cinemotion* is a feasible and promising method for improving these 2 dimensions of social cognition, though more controlled research is needed to confirm the efficacy of this training.

## Author contributions

JS and AnG designed the study procedures and methods, conducted all statistical analyses, interpreted the data and edited subsequent versions of the manuscript. MG and FC collected the data and wrote the first draft of the manuscript. AuG and CM proved advice regarding study procedures, methods, and data interpretation and edited the manuscript several times.

### Conflict of interest statement

The authors declare that the research was conducted in the absence of any commercial or financial relationships that could be construed as a potential conflict of interest.
